# Happiness around the world: A combined etic-emic approach across 63 countries

**DOI:** 10.1371/journal.pone.0242718

**Published:** 2020-12-09

**Authors:** Gwendolyn Gardiner, Daniel Lee, Erica Baranski, David Funder, Members of the International Situations Project

**Affiliations:** 1 Department of Psychology, The University of California, Riverside, California, United States of America; 2 Department of Psychology, The University of Houston, Houston, Texas, United States of America; Sogang University (South Korea), REPUBLIC OF KOREA

## Abstract

What does it mean to be happy? The vast majority of cross-cultural studies on happiness have employed a Western-origin, or “WEIRD” measure of happiness that conceptualizes it as a self-centered (or “independent”), high-arousal emotion. However, research from Eastern cultures, particularly Japan, conceptualizes happiness as including an interpersonal aspect emphasizing harmony and connectedness to others. Following a combined emic-etic approach (Cheung, van de Vijver & Leong, 2011), we assessed the cross-cultural applicability of a measure of independent happiness developed in the US (Subjective Happiness Scale; Lyubomirsky & Lepper, 1999) and a measure of interdependent happiness developed in Japan (Interdependent Happiness Scale; Hitokoto & Uchida, 2015), with data from 63 countries representing 7 sociocultural regions. Results indicate that the schema of independent happiness was more coherent in more WEIRD countries. In contrast, the coherence of interdependent happiness was unrelated to a country’s “WEIRD-ness.” Reliabilities of both happiness measures were lowest in African and Middle Eastern countries, suggesting these two conceptualizations of happiness may not be globally comprehensive. Overall, while the two measures had many similar correlates and properties, the self-focused concept of independent happiness is “WEIRD-er” than interdependent happiness, suggesting cross-cultural researchers should attend to both conceptualizations.

## Introduction

What does it mean to be happy? The answer might depend, at least in part, on cultural context. Laypeople, scientists, and even governments seek to assess the happiness of nations around the world. Some investigators ask which countries have the happiest people, while others seek predictors of happiness at the country or individual level. However, almost all international studies of happiness rely on measures developed in the West, which may impose inappropriate conceptualizations, styles, or values [1–5]. Moreover, empirical research exploring cultural distinctions in happiness beyond just a few countries (usually two)–is sorely lacking. The present article, following a combined etic-emic approach [6], assesses two measures of happiness, developed in the United States and Japan, across 63 countries on all of the inhabited continents of the world.

Early cross-cultural research usually tested the generalizability of established psychological measures, almost always developed in the United States, in other cultures. For example, researchers have assessed the universality of the Big Five personality traits across multiple counties [7, 8]. This method is known as the etic approach. However, the etic approach often overlooks important aspects of a particular culture because they are not included in the original measure, typically developed within Western contexts. The emic approach to cross-cultural psychology attempts to compensate for this problem by developing measures of concepts deemed important to a particular culture, including non-Western contexts, using a bottom-up approach. While the emic approach is crucial for comprehensive assessments of cultural attributes, it often emphasizes cultural uniqueness and lacks widespread applicability outside of the cultural context [6]. The combined etic-emic approach attempts to utilize the benefits of both approaches, by assessing the generalizability of multiple measures of a similar construct across multiple groups in culturally distinctive contexts.

### Cross-cultural research on happiness

The vast majority of research on happiness has originated in WEIRD countries (Western, Educated, Industrialized, Rich, and Democratic [9]), most frequently the United States (while many authors distinguish among terms such as happiness, well-being, positive affect, and life satisfaction, here we incorporate all of these terms under the common construct of happiness for a more comprehensive review of the literature). Accordingly, the prevailing conceptualization of happiness is consistent with a historically Protestant, self-centered worldview that emphasizes personal worthiness and hard work to obtain positive outcomes [10], and sees happiness as a personal achievement rather than the result of good fortune or context [5, 11]. This view further assumes the self is largely independent of others, and thus one’s happiness is independent of others. Additionally, people in Western societies, most notably in America, apparently enjoy higher levels of emotional arousal [12], which may also reflect historical and modern Christian influences [13].

In contrast, the East Asian worldview has been described as one in which the self is more entwined with others, such that personal happiness depends on positive connections in social relationships [5]. For example, one study found that Koreans are more likely than Americans to spontaneously mention the word “family” when asked what they typically associate with the word “happiness” [14]. Additionally, the Eastern view of happiness prioritizes a lower level of emotional arousal [12]. Lower arousal can encompass both positive and negative emotions, with balance and harmony being more valued than a high ratio of positive to negative affect [11, 15].

Previous studies have also found cultural distinctions in predictors and consequences of happiness [16]. Self-esteem is often the strongest predictor of happiness in Western cultures, but this relationship is generally weaker in East Asian cultures [17]. Relational self-esteem, such as being proud of one’s family, is a stronger predictor of subjective well-being for Chinese students than is personal self-esteem [18]. Other predictors of happiness that vary by culture are contextual events, such as positive daily life experiences, which are stronger predictors of well-being for East Asians than for Westerners [19]. Lastly, interventions designed to increase happiness can have different results in different cultures [20]. For example, practicing gratitude is typically associated with increased positive emotions for Americans but may lead to mixed feelings for Koreans, such as feeling guilt or indebtedness along with love [21].

Overall, evidence from cross-cultural studies on the differences in definitions, associations, and consequences of happiness suggests previous Western-centered conceptualizations of happiness are far from universal. Additionally, if the concept of happiness varies cross-culturally, the method of measuring happiness across cultures must also vary accordingly. For example, the Eastern conceptualization of happiness as more intertwined with others may be masked from researchers who only assess happiness using measures developed with a Western, independent focus. Thus, the evidence of cross-cultural differences in happiness point to a greater need for incorporating more culturally sensitive measures of happiness.

### Independent vs. interdependent measures of happiness

Despite the widespread acknowledgment of cultural distinctions in the concept of happiness and the evident need for a measure developed in a non-WEIRD country, emic (indigenous) measures developed outside of the West have become available only recently. One such measure, the Interdependent Happiness Scale (IHS), developed by researchers in Japan [22], was designed to encompass the main components of happiness based on the outlook of individuals in East Asia, specifically Japan. The IHS assesses three main components: relationship orientation, quiescence, and embeddedness in the ordinariness of others. Relationship orientation means that one’s own happiness is dependent upon the happiness of others—an important aspect of this dependency comes from interpersonal harmony. Quiescence comes from an Eastern belief that part of happiness is the absence of negative events or potential for social disruptions that may hinder a peaceful existence. Embeddedness in the ordinariness of others comes from the Eastern preference for normality in the sense that everyone is on an equal level in their success and accomplishments.

The Interdependent Happiness Scale (IHS) differs from traditional Western measures of happiness in both its ideal level of affect and in its lesser emphasis on comparisons with others. For example, one common measure of happiness developed in the West, the Satisfaction with Life Scale (SWLS: [23]), asks individuals how much they agree with the statement “The conditions of my life are excellent,” implying a high level of affect intensity. In contrast, the IHS asks if individuals have “any concerns or anxieties” with the absence of negative affect indicating greater well-being. Likewise, another Western measure of happiness, the Subjective Happiness Scale (SHS: [24]), asks participants to compare themselves to others around them and rate if they are “more happy” or “less happy.” In contrast, the IHS asks participants how much they agree with the statement that they are “just as happy as others around them,” incorporating the interdependence of others’ happiness into the measure. The Western conceptualizations of happiness can be defined in terms of independence while the Eastern conceptualizations of happiness can be defined in terms of interdependence. Thus, from this point forward, we will refer to self-focused, Western conceptualizations of happiness as *independent* happiness and Eastern conceptualizations of happiness as *interdependent* happiness.

Little is known regarding how well these two conceptualizations of happiness generalize beyond the East vs. West dichotomy that seems ubiquitous in cross-cultural research [4]. Non-WEIRD countries encompass a wide range of diverse cultural values, religious beliefs, political institutions, and even geographic conditions that can all influence psychological constructs [25]. These overlapping influences could be expected to affect the extent to which independent or interdependent concepts of happiness generalize cross-culturally. For example, Latin America societies and East Asians societies are both seen as collectivistic, valuing close relationships with others, which would suggest an interdependent view of happiness. However, one study on cultural differences in ideal affect found Mexicans prefer higher arousal positive emotions while Hong Kong Chinese prefer lower arousal positive emotions [26], suggesting the quiescence aspect of the Interdependent Happiness Scale may not apply in Latin American societies. Assessing a wider range of cultures beyond the most commonly included Western and Eastern countries will help further test the generalizability of these two concepts of happiness.

## The current study

The purpose of the present study is to compare and contrast the two cultural conceptualizations of independent and interdependent happiness in many countries around the world. Using a combined etic-emic approach [6], we assessed the Western conceptualization of independent happiness using a measure developed and widely-used in the United States (Subjective Happiness Scale, SHS: [24]) and the Eastern conceptualization of interdependent happiness using the Interdependent Happiness Scale (IHS), developed in Japan [22]. While the Interdependent Happiness Scale (IHS) has been assessed in a number of Eastern and Western countries (e.g., [27]), a large-scale assessment comparing the measure with a Western measure of happiness across diverse cultural contexts has yet to be reported. Additionally, previous cross-cultural research on happiness has typically only compared Westerners (usually in the US or Canada) with East Asians (most commonly Japan), while neglecting cultures in Africa, Latin America, the Middle East, and Southeast Asia [4]. We sought to assess the constructs of Eastern interdependent happiness with a Western measure of independent happiness across a wide range of 63 culturally diverse countries to determine the generalizability of the measures both within and outside of the Eastern and Western contexts.

## Methods

### Participants

Participants (N = 15,368; 71% female) were recruited by local collaborators from 63 countries (see Table 1) and were members of their local university and college communities (*M*_*age*_ = 21.93). The average sample size across all the countries was *n* = 246 (range: 50–1,366). Participants either volunteered or received compensation in the form of extra credit, course credit, small gifts, or monetary payment for participation.

**Table 1:** Demographic information by country.

Country	Region	Total N	% Female	Mean Age
Argentina	Latin America	140	79	24.28
Australia	English West	196	76	19.84
Austria	Europe	113	81	21.26
Belgium	Europe	50	84	19.14
Bolivia	Latin America	135	58	21.01
Brazil	Latin America	310	72	23.69
Bulgaria	Europe	152	70	25.02
Canada	English West	304	79	21.85
Chile	Latin America	386	66	21.47
China	East Asia	432	48	22.63
Colombia	Latin America	181	74	21.68
Croatia	Europe	218	65	21.46
Czech Republic	Europe	193	81	22.65
Denmark	Europe	246	79	22.92
Estonia	Europe	293	84	25.88
France	Europe	231	84	22.58
Georgia	Europe	140	80	20.29
Germany	Europe	458	74	24.36
Greece	Europe	225	80	22.57
Hong Kong	East Asia	144	58	18.99
Hungary	Europe	178	60	21.76
India	South Asia	221	50	22.38
Indonesia	South Asia	131	52	21.83
Israel	Middle East	173	61	25.42
Italy	Europe	717	65	21.86
Japan	East Asia	243	62	22.56
Jordan	Middle East	141	81	19.87
Kenya	Africa	139	65	21.17
Latvia	Europe	169	83	24.87
Lithuania	Europe	145	78	20.26
Macedonia	Europe	54	74	21.22
Malaysia	South Asia	230	70	21.52
Mexico	Latin America	247	58	23.85
Netherlands	Europe	301	81	20.14
New Zealand	English West	129	86	19.19
Nigeria	Africa	135	33	24.72
Norway	Europe	159	74	23.89
Pakistan	South Asia	114	50	20.61
Palestine	Middle East	295	83	22.17
Peru	Latin America	74	61	22.66
Philippines	South Asia	337	68	19.69
Poland	Europe	234	83	22.35
Portugal	Europe	157	87	21.77
Romania	Europe	177	57	22.84
Russia	Europe	159	78	21.90
Senegal	Africa	635	47	23.31
Serbia	Europe	185	86	19.72
Singapore	South Asia	136	78	20.93
Slovakia	Europe	148	70	22.41
Slovenia	Europe	123	57	20.59
South Africa	Africa	256	66	22.20
South Korea	East Asia	281	58	22.35
Spain	Europe	419	85	19.73
Sweden	Europe	130	70	†
Switzerland	Europe	755	84	22.35
Taiwan	East Asia	162	77	19.71
Thailand	South Asia	196	77	19.27
Turkey	Middle East	329	68	21.09
Uganda	Africa	93	65	22.63
Ukraine	Europe	244	77	20.62
United Kingdom	Europe	136	89	25.64
United States	English West	1366	67	19.86
Vietnam	South Asia	168	77	19.05
World Average		246	71	21.93

Note

† = Data not available.

### Measures

The analyses presented below stem from the International Situations Project (ISP), a large cross-cultural study assessing situational experience, daily behavior, and individual differences. Other analyses based on this large and diverse data set have been published [28–30] or are in progress, but all analyses reported in the present article are new and unique. For an overview of the project, including all measures and translations, see situationslab.com/the-international-situations-project. Only measures included in the present analyses are described in this article, along with country-level variables collected previously and separately by other researchers or obtained from public databases.

#### Independent happiness

The Western measure of happiness was the Subjective Happiness Scale (SHS: [24]). The SHS is one of the most widely used measures of happiness in studies conducted in the US and Europe. The measure has 4 items to which participants respond on a 7-point scale (e.g., “Compared with most of my peers, I consider myself…” 1 = *less happy* to 7 = *more happy*).

#### Interdependent happiness

The Eastern measure of happiness was the Interdependent Happiness Scale [22]. The IHS was developed in Japan and validated against samples in the United States, Germany, and South Korea. The measure has 9 items to which participants respond on a 5-point scale (e.g., “I believe that my life is just as happy as that of others around me” 1 = *strongly disagree*, 5 = *strongly agree*).

#### Country-level variables

The current analyses use several country-level variables obtained from publicly available databases. Country level variables were chosen to represent a range of socioecological, geographic, and psychological variables that could be feasibly related to country level differences in the conceptualizations of happiness [25, 31]. We grouped the country level variables into two categories of “objective” variables (statistics measured by government or other organizations) and “subjective” variables (aggregated from individual responses to psychological measurements). For a complete list of all country scores for each of the listed variables, see S1 File.

*Objective country-level variables*. A number of “objective” country level variables were chosen to reflect basic characteristics of the country as measured by various organizations, selected based on plausible relevance to happiness and the availability of data for at least 40 of the countries included in our data. These variables were also chosen to be as independent from each other as possible, as many country characteristics (e.g., GDP & life expectancy) are highly correlated with each other and would thus produce redundant results.

*Human Development Index (HDI)*. The Human Development Index (HDI) is a composite measure of a country’s development, consisting of life expectancy, educational opportunities, and standard of living [32]. HDI scores were available for all ISP countries except Taiwan. Country HDI scores ranged from .49 (Uganda & Senegal) to .95 (Norway), with higher scores indicating greater economic development.

*Population density*. Population density is the number of people per sq. km of land area (The World Bank [33]). Population density data was available for all ISP countries except Taiwan; however, both Hong Kong (7,040 people per sq. km) and Singapore (7,916 people per sq. km) were excluded from analyses because their unusually high density skewed the country-level results. The remaining population density scores ranged from 3 people per sq. km (Australia) to 756 people per sq. km (Palestine).

*Growth rate*. Population growth rate is the average annual percent change in population of a country [34]. Growth rate data was available for all ISP countries except Palestine. Country scores ranged from -1.08 (Latvia) to 3.20 (Uganda), with positive scores indicating an increase in population size and negative scores indicating a decrease in population size.

*Suicide rate*. Suicide rate is the age-standardized suicide rate per 100,000 people, averaged across sexes [35]. Suicide rates were available for 60 ISP countries. Country scores ranged from 2.50 (Pakistan) to 26.10 (Lithuania), with high scores indicating a higher suicide rate.

*Average temperature*. Average temperature is the average daily temperature throughout the entire year in Celsius [36]. Because some larger countries have a wide range of average temperatures depending upon exact location, the average temperature used was that of the city or cities in which ISP data collection took place. For most countries, only one city was included in the average daily temperature. Data on average daily temperature was available for all 63 ISP countries and ranged from 4°C (Russia) to 29°C (Thailand).

*Subjective country level variables*. Subjective country level variables were chosen to reflect the psychological or cultural characteristics of a country.

*WEIRDness*. *WEIRD* country level scores are a measure of cultural distance from the United States [37]. As computed by Muthukrishna and colleagues [37], the scores reflect a country’s overall dissimilarity to the United States on a range of psychological variables from the World Values Survey (WVS), including personality traits, cultural values, and tightness/looseness. These psychological variables were selected by the authors to include all questions from the WVS that were judged to be culturally transmissible. The United States was chosen as the reference group because of the large American dominance in the field of psychology. Psychological distance scores were also calculated for China as a comparison, but were excluded from present analyses because the comparison measure of interdependent happiness was developed in Japan. Notably, the cultural distance calculated between the United States and Japan was similar to the cultural distance between China and Japan, meaning the scores computed for China would not be representative of the cultural similarity to the IHS. For the cultural distance scores presented for the United States, we reversed the country scores to make higher scores indicate more similarity and thus a higher level of “WEIRD-ness” as it was originally conceptualized (i.e., more Western, Educated, Industrialized, Rich and Democratic, similar to the United States). Cultural distance WEIRD scores were available for 46 ISP countries. The ‘most WEIRD’ countries (most psychologically similar to the United States) were Canada (.97) and Australia (.97), and the least WEIRD countries was Jordan (.81).

*Cultural values*. Schwartz’s cultural value orientation scales represent seven distinct bipolar values assessed in national surveys of students and teachers in 80 countries [38]. The scales measure *embeddedness* (how embedded people are in their groups), *intellectual autonomy* (the independent pursuit of ideas and knowledge), *affective autonomy* (the independent pursuit of pleasure), *harmony* (valuing the group rather than the self), *egalitarianism* (valuing cooperation and concern for all), *hierarchy* (reliance on structured and hierarchical social roles), and *mastery* (valuing success through self-assertion). Country scores for all seven of Schwartz’s cultural values were available for 59 ISP countries.

### Procedure

Local collaborators (all of whom were psychologists) translated each of the measures into their local language, which were then back translated into English by an independent translator. The original English version was then compared with the back-translated measure and discrepancies were resolved. This method was used to translate all of the research materials into 42 languages. The local collaborators then recruited participants from their college communities (largely students) to log on to our custom-built website (ispstudy.net) with a unique participant ID. They then completed the informed consent process followed by a series of measures, including the happiness measures reported here. Upon completing the survey, participants had the opportunity to receive feedback on their personality trait levels based on their ratings on the personality measure included in the survey (a complete wireframe of the study’s website is available online at https://osf.io/jrbt3/). All procedures were in accordance with the ethical standards of the University of California, Riverside, Office of Research Integrity, who approved this study (HS-11-046), and with the 1964 Helsinki declaration and its later amendments or comparable ethical standards.

### Data analytic strategy

Data analyses were separated into those at the individual level and country level to assess how the performance of the happiness measures vary cross-culturally. The first set of analyses were conducted at the individual level, within each country, and results are presented for all 63 countries. These individual level analyses include several internal consistency tests including general factor saturation (*ω*_*h*_) and total common variance (*ω*_*t*_) [39]. Additionally, because the two happiness measures have an unequal number of items, we present the average communality score (${\bar{h}}^{2}$) and the smallest split half reliability (*β*) score for each measure. These tests of reliability were all conducted separately within each country and then averaged within geographic and cultural regions. To test for the association between the two happiness measures within each country, we used Structural Equation Modeling (SEM) to account for measurement error. Readers interested in comparing the mean levels of the happiness measures across countries may reference the S1 File, but those scores were not included in any of the present analyses.

Because results are presented for many countries here, the second set of analyses attempts to find patterns in the data by analyzing relationships between variables on the *country level*. Specifically, what country level variables are associated with higher or lower reliability of the happiness measures. These country level tests use the individual level analyses presented within each country as well as country level data collected independently from the current study, to reduce method bias [40]. Given the potential for spurious country-level correlations due to the high number of potential relationships being tested and the subjective manner in which external country variables were selected, randomization tests determined the number of relationships expected by chance [41]. Out of a total of 117 possible correlations (9 averaged individual level values, 13 external country level values) about 7 were expected to be significant by chance. The number of observed statistically significant correlations in the data is 44 (*p* < .001), with an average absolute *r* = .25 (*p* < .001, expected average absolute *r* = .13).

Both individual and country level analyses were conducted in R using the *psych* [42], *multicon* [43], and *lavaan* [44] packages. All data and R code necessary to recreate the analyses presented here are available on the OSF project page (https://osf.io/jrbt3/).

## Results

### Individual level happiness measure analyses within each country

#### Reliability of the happiness measures

The first set of individual level analyses concern the reliability of the happiness measures within each country. We present multiple tests of internal consistency using the broad approach prescribed by generalizability theory [39]. Each result is presented for each country, considering each as a separate sample, as well as the average across all countries. The internal consistency scores for the Subjective Happiness Scale (SHS) are presented in Table 2 and the internal consistency scores for the Interdependent Happiness Scale (IHS) are presented in Table 3. Averages of these countries’ scores for both happiness measures within geographic regions are presented in Table 4 (see Table 1 for a list of countries and their corresponding region).

**Table 2:** Reliability measures of the Subjective Happiness Scale (SHS) by country.

Country	*ω*_*t*_	*ω*_*h*_	*β*	${\bar{h}}^{2}$
Argentina	.83	.06	.82	.56
Australia	.89	.83	.83	.68
Austria	.86	.83	.83	.63
Belgium	.93	.91	.91	.78
Bolivia	.87	.00	.86	.63
Brazil	.86	.83	.83	.61
Bulgaria	.92	.82	.83	.77
Canada	.89	.87	.87	.69
Chile	.89	.86	.86	.68
China	.83	.04	.78	.57
Colombia	.77	.38	.66	.50
Croatia	.91	.86	.86	.73
Czech Republic	.90	.84	.84	.71
Denmark	.91	.88	.88	.73
Estonia	.88	.00	.87	.65
France	.89	.79	.79	.70
Georgia	.80	.78	.73	.53
Germany	.91	.87	.86	.72
Greece	.85	.82	.81	.60
Hong Kong	.82	.80	.74	.56
Hungary	.86	.82	.82	.62
India	.65	.62	.60	.35
Indonesia	.74	.30	.34	.54
Israel	.76	.07	.70	.50
Italy	.86	.83	.82	.62
Japan	.84	.79	.75	.60
Jordan	.75	.72	.64	.49
Kenya	.72	.01	.66	.43
Latvia	.92	.84	.84	.76
Lithuania	.89	.33	.85	.69
Macedonia	.84	.77	.77	.60
Malaysia	.71	.01	.59	.44
Mexico	.78	.01	.72	.49
Netherlands	.92	.88	.87	.76
New Zealand	.86	.01	.83	.62
Nigeria	.74	.48	.58	.48
Norway	.89	.85	.85	.68
Pakistan	.68	.37	.39	.48
Palestine	.70	.01	.58	.42
Peru	.90	.88	.88	.71
Philippines	.83	.08	.79	.57
Poland	.90	.86	.85	.70
Portugal	.88	.79	.79	.67
Romania	.86	.30	.79	.64
Russia	.87	.85	.85	.64
Senegal	.59	.54	.46	.31
Serbia	.89	.79	.81	.69
Singapore	.89	.85	.83	.68
Slovakia	.86	.78	.81	.63
Slovenia	.87	.83	.83	.64
South Africa	.88	.86	.85	.66
South Korea	.91	.84	.86	.72
Spain	.89	.84	.84	.68
Sweden	.91	.89	.89	.73
Switzerland	.87	.83	.83	.64
Taiwan	.88	.02	.86	.67
Thailand	.89	.02	.86	.67
Turkey	.87	.84	.83	.64
Uganda	.69	.20	.20	.49
Ukraine	.82	.42	.75	.57
United Kingdom	.94	.86	.88	.80
United States	.87	.84	.82	.64
Vietnam	.74	.04	.65	.46
**Average**	**.84**	**.59**	**.77**	**.62**
SD	.08	.34	.14	.11

*Note*. ω_*t*_ = total common variance, ω_*h*_ = general factor saturation, β = smallest split half reliability, ${\bar{h}}^{2}$ = average communality score.

**Table 3:** Reliability measures of the Interdependent Happiness Scale (IHS) by country.

Country	*ω*_*t*_	*ω*_*h*_	*β*	${\bar{h}}^{2}$
Argentina	.81	.56	.58	.44
Australia	.82	.57	.64	.45
Austria	.78	.60	.55	.41
Belgium	.81	.48	.57	.46
Bolivia	.85	.59	.66	.49
Brazil	.84	.69	.65	.47
Bulgaria	.88	.63	.74	.54
Canada	.85	.66	.68	.47
Chile	.87	.74	.73	.50
China	.88	.73	.80	.49
Colombia	.86	.57	.67	.50
Croatia	.84	.60	.65	.46
Czech Republic	.82	.52	.57	.46
Denmark	.85	.61	.65	.49
Estonia	.82	.58	.65	.44
France	.83	.51	.57	.49
Georgia	.83	.55	.62	.46
Germany	.82	.60	.66	.43
Greece	.81	.44	.56	.42
Hong Kong	.88	.61	.76	.53
Hungary	.80	.49	.61	.41
India	.79	.62	.64	.38
Indonesia	.77	.50	.54	.41
Israel	.87	.50	.64	.52
Italy	.80	.54	.55	.44
Japan	.86	.62	.71	.48
Jordan	.89	.59	.69	.57
Kenya	.82	.42	.46	.50
Latvia	.83	.65	.56	.50
Lithuania	.86	.58	.69	.50
Macedonia	.81	.49	.48	.48
Malaysia	.85	.67	.69	.47
Mexico	.83	.59	.63	.47
Netherlands	.84	.67	.67	.46
New Zealand	.89	.74	.77	.55
Nigeria	.86	.52	.60	.52
Norway	.85	.65	.63	.50
Pakistan	.77	.52	.59	.36
Palestine	.83	.64	.59	.45
Peru	.90	.70	.69	.58
Philippines	.85	.59	.66	.48
Poland	.84	.64	.62	.47
Portugal	.81	.32	.63	.44
Romania	.85	.64	.69	.47
Russia	.82	.60	.63	.46
Senegal	.82	.55	.59	.44
Serbia	.89	.64	.73	.56
Singapore	.85	.62	.69	.47
Slovakia	.89	.70	.75	.54
Slovenia	.83	.58	.63	.44
South Africa	.84	.64	.64	.46
South Korea	.89	.75	.79	.55
Spain	.84	.66	.71	.46
Sweden	.89	.63	.68	.57
Switzerland	.82	.56	.61	.44
Taiwan	.85	.77	.67	.48
Thailand	.89	.81	.76	.57
Turkey	.83	.63	.63	.44
Uganda	.74	.41	.47	.37
Ukraine	.80	.54	.56	.43
United Kingdom	.85	.65	.72	.46
United States	.84	.68	.69	.44
Vietnam	.84	.65	.69	.47
**Average**	**.84**	**.60**	**.64**	**.47**
SD	.03	.09	.07	.05

*Note*. ω_*t*_ = total common variance, ω_*h*_ = general factor saturation, β = smallest split half reliability, ${\bar{h}}^{2}$ = average communality score.

**Table 4:** Reliability measures for the Subjective Happiness Scale (SHS) and Interdependent Happiness Scale (IHS) averaged by region.

Region	SHS *ω*_*t*_	IHS *ω*_*t*_	SHS *ω*_*h*_	IHS *ω*_*h*_	SHS *β*	IHS *β*	SHS ${\bar{h}}^{2}$	IHS ${\bar{h}}^{2}$	IHSxSHS
West English	.88	.85	.64	.66	.84	.70	.66	.48	.66
Western Europe	.90	.83	.85	.58	.85	.64	.71	.47	.79
Eastern Europe	.87	.83	.64	.59	.82	.63	.65	.46	.85
Southern Europe	.88	.84	.82	.56	.82	.62	.67	.48	.74
Latin America	.84	.85	.43	.63	.80	.66	.60	.49	.82
East Asia	.86	.87	.50	.70	.80	.75	.62	.51	.70
South Asia	.77	.83	.29	.62	.63	.66	.52	.45	.81
Middle East	.77	.86	.41	.59	.69	.64	.51	.50	.83
Africa	.72	.81	.42	.51	.55	.55	.48	.46	.85
**Average**	.83	.84	.56	.60	.76	.65	.60	.48	.78

*Note*. ω_*t*_ = total common variance, ω_*h*_ = general factor saturation, β = smallest split half reliability, ${\bar{h}}^{2}$ = average communality score.

*Total common variance (ω*_*t*_*)*. We first estimated the total reliability of the happiness measures using McDonald’s [45] omega total (*ω*_*t*_*)*. This metric is similar to Cronbach’s alpha, and can be interpreted along the same scale, but provides a better estimate of reliability [39]. Both the SHS and the IHS had identical average total common variance across countries (*ω*_*t Mean*_ = .84). For the SHS, only four countries had *ω*_*t*_ *<* .*70*: Senegal (*ω*_*t*_ = .59), India (*ω*_*t*_ = .65), Pakistan (*ω*_*t*_ = .68), and Uganda (*ω*_*t*_ = .69). The countries with the highest SHS total common variance were the United Kingdom (*ω*_*t*_ = .94) and Belgium (*ω*_*t*_ = .93). Overall, countries in Africa had the lowest total variance (*ω*_*t Mean*_ = .72) while Western Europe had the highest (*ω*_*t Mean*_ = .90). For the IHS, none of the countries had a total common variance score *ω*_*t*_ *<* .*70*. The countries with the lowest total common variance were Uganda (*ω*_*t*_ = .74) and Indonesia (*ω*_*t*_ = .77) while the highest proportion was in Peru (*ω*_*t*_ = .90). Similar to the SHS, the lowest total common variance for the IHS was found in African countries (*ω*_*t Mean*_ = .81) but the highest proportions were in East Asian countries (*ω*_*t Mean*_ = .87). While the total reliability for both happiness measures were lowest in African countries, the average was higher for the IHS *ω*_*t (Mean*_ = .81) than the SHS (*ω*_*t Mean*_ = .72).

*General factor saturation (ω*_*h*_*)*. Next, we estimated the proportion of the variance in the observed happiness scores that can be attributed to the general latent factor. The general factor saturation of the test was calculated using McDonald’s [45] omega hierarchical (*ω*_*h*_) coefficient. Omega hierarchical is a useful test for assessing the homogeneity of a measure. A low score would indicate that the observed scores are not accurate predictors of the latent score and the variability in the items may be due to other factors [39]. Omega hierarchical is useful because, unlike omega total, the reliability estimates are not a function of test length. This is particularly important when comparing the reliability of two measures with unequal numbers of items, as is the case for the two measures of happiness. Both the SHS (*ω*_*hMean*_ = .59) and IHS (*ω*_*hMean*_ = .60) average coefficients were very similar, however the SHS (*ω*_*hSD*_ = .34) varied considerably more than the IHS (*ω*_*hSD*_ = .08). The countries with the lowest SHS general factor saturation coefficients were Bolivia (*ω*_*h*_ = .002) and Estonia (*ω*_*h*_ = .003) while the countries with the highest SHS general factor saturation were Belgium (*ω*_*h*_ = .91) and Sweden (*ω*_*h*_ = .89). The region with the highest average SHS general factor saturation was Western Europe (*ω*_*hMean*_ = .85) while the lowest scores were found in South Asian countries (*ω*_*hMean*_ = .29). For the IHS, the countries with the lowest general factor saturation were Portugal (*ω*_*h*_ = .32) and Uganda (*ω*_*h*_ = 41) while the highest countries were Thailand (*ω*_*h*_ = .81) and Taiwan (*ω*_*h*_ = .77). The region with the highest average IHS general factor saturation was East Asia (*ω*_*hMean*_ = .70) while African countries had the lowest average (*ω*_*hMean*_ = .51).

*Smallest split half reliability (β)*. Another assessment of the homogeneity of a test is the smallest split half reliability of the test, calculated from all possible splits of the items for each happiness measure The smallest split half reliability is similar to an alpha or *ω*_*t*_, as it is an estimate of the total reliable variance. However, similar to *ω*_*h*_, it is not influenced by test length, and thus useful for comparing measures with unequal items. For interpreting results, a *β* around .50 would indicate that about half of test reflects one general factor of happiness [39]. The SHS had the highest averaged smallest split half reliability (*β*_*Mean*_ = .77) than the IHS (*β*_*Mean*_ = .64). The worst lowest split half reliability for the SHS was in Uganda (*β* = .20), followed by Indonesia (*β* = .34) and Pakistan (*β* = .39). The best lowest split half reliability scores for the SHS were in Belgium (*β* = .91) and Sweden (*β* = .89). Overall, for SHS, the worst lowest split half reliabilities were in African countries (*β*_*Mean*_ = .55) while the best lowest split half reliabilities were in Western European countries (*β*_*Mean*_ = .85) and Western English-speaking countries (*β*_*Mean*_ = 84). For the IHS, the worst lowest split half reliability was in Kenya (*β* = .46) followed by Uganda (*β* = .47) while the best lowest split half reliability scores were in China (*β* = .80) and South Korea (*β* = .79). Similar to the SHS, the worst lowest split half reliability scores for the IHS were in African countries (*β*_*Mean*_ = .55) but the best lowest split half reliability scores were in East Asian countries (*β*_*Mean*_ = .75).

*Communality scores* (${\bar{h}}^{2}$). Communality scores are the square of the factor loadings of the item on the latent trait and represent the percent of variance in the item that can be explained by the latent trait [46]. As communality scores are essentially correlation coefficients, the results can be interpreted similarly [47], with scores of less than .40 suggesting the items may not be strongly related to the latent variable. Tables 5 and 6 present the communality scores for the SHS and IHS across countries, respectively. Because the two happiness measures do not have an equal number of items, we also calculated the average communality score for each measure [39], presented in Table 4.

**Table 5:** Communality scores (${\bar{h}}^{2}$) for the Subjective Happiness Scale (SHS).

Country	Item #1 ${\bar{h}}^{2}$	Item #2 ${\bar{h}}^{2}$	Item #3 ${\bar{h}}^{2}$	Item #4 ${\bar{h}}^{2}$	Average ${\bar{h}}^{2}$
Argentina	.63	.65	.50	.46	.56
Australia	.84	.75	.73	.40	.68
Austria	.46	.79	.63	.64	.63
Belgium	.69	.79	.87	.75	.78
Bolivia	.81	.67	.56	.47	.63
Brazil	.69	.72	.68	.36	.61
Bulgaria	.84	.78	.84	.63	.77
Canada	.69	.76	.75	.56	.69
Chile	.69	.80	.73	.51	.68
China	.73	.76	.63	.14	.57
Colombia	.72	.74	.42	.12	.50
Croatia	.77	.79	.77	.60	.73
Czech Republic	.83	.76	.70	.55	.71
Denmark	.77	.83	.73	.59	.73
Estonia	.83	.70	.61	.48	.65
France	.76	.75	.77	.53	.70
Georgia	.72	.74	.41	.25	.53
Germany	.83	.78	.68	.60	.72
Greece	.67	.75	.65	.34	.60
Hong Kong	.82	.78	.44	.20	.56
Hungary	.66	.75	.59	.50	.62
India	.41	.45	.32	.22	.35
Indonesia	.79	.62	.49	.28	.54
Israel	.55	.62	.68	.15	.50
Italy	.74	.75	.58	.39	.62
Japan	.77	.76	.46	.40	.60
Jordan	.70	.62	.53	.13	.49
Kenya	.59	.50	.59	.05	.43
Latvia	.87	.86	.76	.56	.76
Lithuania	.79	.85	.80	.34	.69
Macedonia	.72	.83	.40	.46	.60
Malaysia	.61	.74	.39	.03	.44
Mexico	.56	.80	.45	.15	.49
Netherlands	.80	.77	.74	.71	.76
New Zealand	.87	.74	.59	.28	.62
Nigeria	.78	.58	.28	.28	.48
Norway	.77	.76	.70	.49	.68
Pakistan	.87	.64	.38	.02	.48
Palestine	.69	.64	.32	.03	.42
Peru	.81	.76	.71	.58	.71
Philippines	.75	.67	.62	.24	.57
Poland	.80	.81	.70	.48	.70
Portugal	.78	.75	.59	.57	.67
Romania	.74	.72	.80	.29	.64
Russia	.74	.71	.67	.46	.64
Senegal	.46	.46	.22	.11	.31
Serbia	.90	.79	.64	.42	.69
Singapore	.87	.85	.60	.40	.68
Slovakia	.67	.70	.78	.37	.63
Slovenia	.79	.77	.52	.48	.64
South Africa	.77	.80	.70	.37	.66
South Korea	.83	.81	.74	.50	.72
Spain	.70	.73	.67	.64	.68
Sweden	.80	.82	.75	.54	.73
Switzerland	.74	.70	.65	.48	.64
Taiwan	.67	.91	.76	.33	.67
Thailand	.85	.67	.73	.43	.67
Turkey	.74	.75	.61	.47	.64
Uganda	.75	.70	.29	.23	.49
Ukraine	.78	.72	.55	.22	.57
United Kingdom	.85	.88	.78	.70	.80
United States	.78	.77	.68	.32	.64
Vietnam	.79	.60	.40	.07	.46
**Average**	**.74**	**.73**	**.61**	**.39**	**.62**

**Table 6:** Communality scores (${\bar{h}}^{2}$) for the Subjective Happiness Scale (SHS).

Country	#1 ${\bar{h}}^{2}$	#2 ${\bar{h}}^{2}$	#3 ${\bar{h}}^{2}$	#4 ${\bar{h}}^{2}$	#5 ${\bar{h}}^{2}$	#6 ${\bar{h}}^{2}$	#7 ${\bar{h}}^{2}$	#8 ${\bar{h}}^{2}$	#9 ${\bar{h}}^{2}$	Avg ${\bar{h}}^{2}$
Argentina	.42	.28	.34	.22	.20	.28	.56	.65	1.00	.44
Australia	.29	.48	.81	.21	.68	.15	.57	.37	.51	.45
Austria	.35	.17	.22	.08	.40	1.00	.61	.41	.46	.41
Belgium	.29	1.00	.36	.16	.13	.26	.30	.64	1.00	.46
Bolivia	.83	.40	.39	.32	.32	.37	.59	.71	.48	.49
Brazil	.50	.30	.49	.26	.24	1.00	.63	.45	.40	.47
Bulgaria	.53	.63	.36	.30	.39	1.00	.59	.59	.49	.54
Canada	.33	.67	.38	.35	.45	.30	.57	.40	.82	.47
Chile	.48	.30	.43	.35	1.00	.23	.69	.53	.53	.50
China	.39	.58	.41	.47	.34	.54	.55	.40	.69	.49
Colombia	.58	.22	1.00	.33	.43	.40	.62	.46	.47	.50
Croatia	.32	.42	.40	.44	.49	.32	.57	.47	.70	.46
Czech Republic	.32	.11	1.00	.16	.61	.29	.51	.42	.73	.46
Denmark	.42	.36	.41	.50	.58	.23	.82	.44	.68	.49
Estonia	.39	.42	.45	.10	.74	.30	.39	.50	.65	.44
France	.39	.34	.33	.24	1.00	.25	.69	.53	.60	.49
Georgia	.40	.21	.33	.42	.77	.21	.47	.67	.63	.46
Germany	.31	.42	.66	.11	.69	.27	.46	.32	.66	.43
Greece	.35	.31	.39	.57	.21	.22	.68	.40	.67	.42
Hong Kong	.42	.57	.40	.41	.50	.33	.62	1.00	.52	.53
Hungary	.36	.48	.46	.08	.44	.39	.38	.38	.73	.41
India	.28	.39	.58	.20	.43	.21	.58	.36	.38	.38
Indonesia	.32	.90	.47	.17	.04	.27	1.00	.22	.28	.41
Israel	.52	.77	.65	.34	.29	.30	.76	.67	.42	.52
Italy	.44	.41	.33	.01	1.00	.14	.61	.48	.52	.44
Japan	1.00	.51	.29	.42	.28	.19	.63	.52	.53	.48
Jordan	.54	.54	.61	.51	1.00	.28	.56	.53	.61	.57
Kenya	.31	.30	.40	.40	1.00	.39	.56	.54	.56	.50
Latvia	.23	.13	1.00	.27	.33	.67	.61	.47	.79	.50
Lithuania	.43	.50	.40	.16	1.00	.24	.50	.54	.77	.50
Macedonia	1.01	.07	.09	.84	.22	.14	.52	.64	.80	.48
Malaysia	.52	.37	.29	.54	.32	.16	.58	1.00	.44	.47
Mexico	.95	.25	.25	.34	.20	.43	.63	.51	.65	.47
Netherlands	.45	.35	.71	.18	.50	.17	.72	.49	.55	.46
New Zealand	.49	1.00	.16	.26	.35	.59	.77	.63	.72	.55
Nigeria	.43	.39	.51	.39	.15	1.00	.60	.60	.57	.52
Norway	.27	.87	.32	.27	.59	.24	.68	.61	.62	.50
Pakistan	.28	.51	.28	.20	.18	.33	.63	.41	.45	.36
Palestine	.47	.23	.50	.39	.44	.23	.40	.56	.80	.45
Peru	.73	.40	.54	.40	.54	.46	.76	.74	.63	.58
Philippines	.33	.50	.48	.21	.74	.27	.57	.64	.61	.48
Poland	.33	1.00	.20	.36	.52	.22	.66	.46	.49	.47
Portugal	.37	.48	1.00	.36	.20	.21	.55	.54	.26	.44
Romania	.47	.50	.41	.33	.53	.24	.56	.55	.70	.47
Russia	.29	.47	.30	.12	1.00	.28	.82	.38	.48	.46
Senegal	.35	.39	.33	.37	.22	1.00	.36	.48	.51	.44
Serbia	.53	.45	.53	.33	1.00	.20	.58	.62	.80	.56
Singapore	.36	.43	.44	.58	.31	.32	.64	.47	.69	.47
Slovakia	.38	.71	.51	.37	.52	.31	.61	.64	.76	.54
Slovenia	.47	.69	.06	.34	.46	.22	.55	.56	.62	.44
South Africa	.35	.28	.60	.32	.47	.25	.59	.50	.81	.46
South Korea	.58	.38	.38	.41	.43	1.00	.64	.59	.54	.55
Spain	.46	.33	.36	.24	1.00	.18	.60	.34	.67	.46
Sweden	.33	.69	.54	.46	.60	.40	.54	.56	1.00	.57
Switzerland	.30	.45	.36	.17	.84	.18	.56	.47	.61	.44
Taiwan	.42	.35	1.00	.24	.30	.26	.69	.38	.66	.48
Thailand	.35	.90	.30	.44	1.00	.28	.73	.53	.59	.57
Turkey	.29	.36	.29	.19	.66	.33	.63	.48	.70	.44
Uganda	.50	.36	.11	.33	.59	.13	.38	.68	.27	.37
Ukraine	.39	.58	.35	.18	.46	.26	.41	.50	.71	.43
United Kingdom	.32	.33	.79	.20	.55	.27	.56	.48	.68	.46
United States	.44	.54	.22	.21	.57	.28	.55	.52	.58	.44
Vietnam	1.00	.61	.41	.24	.27	.20	.60	.31	.60	.47
**Average**	**.44**	**.47**	**.45**	**.31**	**.52**	**.35**	**.59**	**.52**	**.62**	**.47**

The bottom row of Table 5 presents the average communality score for each item of the Subjective Happiness Scale across countries. The first 3 items of the SHS had high communality scores (ranging from .60 to .70), suggesting a high proportion of their variability could be explained by the latent independent happiness variable. However, there was a substantial drop in communality scores for the fourth item on the scale. The communality score for the SHS item #4 was less than .40, suggesting this item may not be as strongly related as the other items. Notably, item #4 is also the only reversed item on the scale–“Some people are generally not very happy…To what extent does this characterize you?”. For some countries, such as Kenya, Vietnam, and Pakistan, the communality scores for the first three items were all acceptable while the communality score for item #4 was almost zero. Even in the United States, the country of origin for the measure, the communality score for item #4 might not be considered acceptable. Overall, this suggests this item should be removed to improve the overall reliability of the measure.

For the Interdependent Happiness Scale, the communality scores for all of the items were much more consistent. Two of the items (#4 & #6) had average communality scores below .40 but were not substantially lower than the other items that ranged from .40 to .60. These two items from the IHS pertain to the quiescence component of the scale, regarding the absence of negative aspects in one’s life. However, while these two items were lowest on average, these items were not consistently low within countries. For example, Austria and Brazil had low (< .30) communality scores for item #4 but extremely high communality scores for #6. However, in Japan, the country of origin for the IHS, the communality score for item #6 was considerably lower. The item with the highest overall average communality score was #9, “I generally believe that things are going well for me in its own way as they are for others around me,” followed by items #7 and #8. These last three items on the measure pertain to the embeddedness aspect of interdependent happiness.

Each measure’s average communality score was calculated as the average of each item’s communality score within each country and then averaged across countries (see Table 4). Across all countries, the average communality scores for the SHS (${\bar{h}}^{2}{}_{\mathrm{Mean}}$ = .62) were higher than the average communality scores for the IHS (${\bar{h}}^{2}{}_{\mathrm{Mean}}$ = .47). The countries with the lowest average communality scores for the SHS were Senegal (${\bar{h}}^{2}$ = .31) and India (${\bar{h}}^{2}$ = .35), while the highest scores were in the United Kingdom (${\bar{h}}^{2}$ = .80) and Belgium (${\bar{h}}^{2}$ = .78). Overall, the lowest average communality scores for the SHS were in Africa (${\bar{h}}^{2}{}_{\mathrm{Mean}}$ = .48) while the highest average communality scores were in Western Europe (${\bar{h}}^{2}{}_{\mathrm{Mean}}$ = .71). For the IHS, the countries with the lowest average communality scores were Pakistan (${\bar{h}}^{2}$ = .36) and Uganda (${\bar{h}}^{2}$ = .37) while the highest average communality scores were in Peru (${\bar{h}}^{2}$ = .58) and Jordan, Sweden, and Thailand (${\bar{h}}^{2}$ = .57). Overall, the lowest average communality scores for the IHS were in South Asia (${\bar{h}}^{2}{}_{\mathrm{Mean}}$ = .45) and the best average communality scores were in East Asia (${\bar{h}}^{2}{}_{\mathrm{Mean}}$ = .51).

#### Relationship between happiness measures

To test for the relationship between the two happiness measures we used Structural Equation Modeling (SEM) to account for differences in the reliability of the measures. For the Interdependent Happiness Scale (IHS), the 9 items were grouped into 3 corresponding parcels to decrease the total number of parameters estimated. There were no missing data and thus no imputation was needed.

Given the range of sample sizes across countries, post hoc power analyses were conducted for estimating the relationship between the two latent variables using the *pwrSEM* app [48]. Rather than calculate power estimates for all 63 countries, we tested the power to detect an effect given the average observed relationships among variables and then with a combination of the lowest observed relationships among variables. For the first power analysis, we estimated the factor loadings for the 4 item SHS should be .75, given an average reliability of .84. The factor loadings for the 3 item IHS with an average reliability of .84 were estimated at .80. The average correlation between the observed SHS and IHS in the data was r = .59, which gives an estimated latent variable correlation of .69. Given these estimated parameters and an average sample size of 246 participants across countries, we estimated the power to detect an effect between the two latent happiness variables to approach 1.

Next, we conducted a power analyses using the lowest observed values, to determine the minimum power we could expect for any of our countries. The lowest reliability of the SHS was .59 (Senegal), so the estimated factor loadings were set to .51. For the IHS, the lowest reliability observed was .74 (Uganda), so the estimated factor loadings were set to .70. The smallest observed correlation between the two happiness measures was r = .26 (Indonesia), so using the lowest reliabilities we estimated the lowest correlation between the two latent variables to be .39. Lastly, power was calculated using these parameter estimates with the smallest sample in our data of 54 (Macedonia), resulting in power of .72 to detect an effect between the happiness measures. Given that there would still be reasonable power to detect an effect despite this exact combination of lowest possible parameters not actually appearing in our data, we concluded all of our country’s sample sizes were sufficient for estimating the latent relationship between the SHS and IHS.

**Fig 1:**
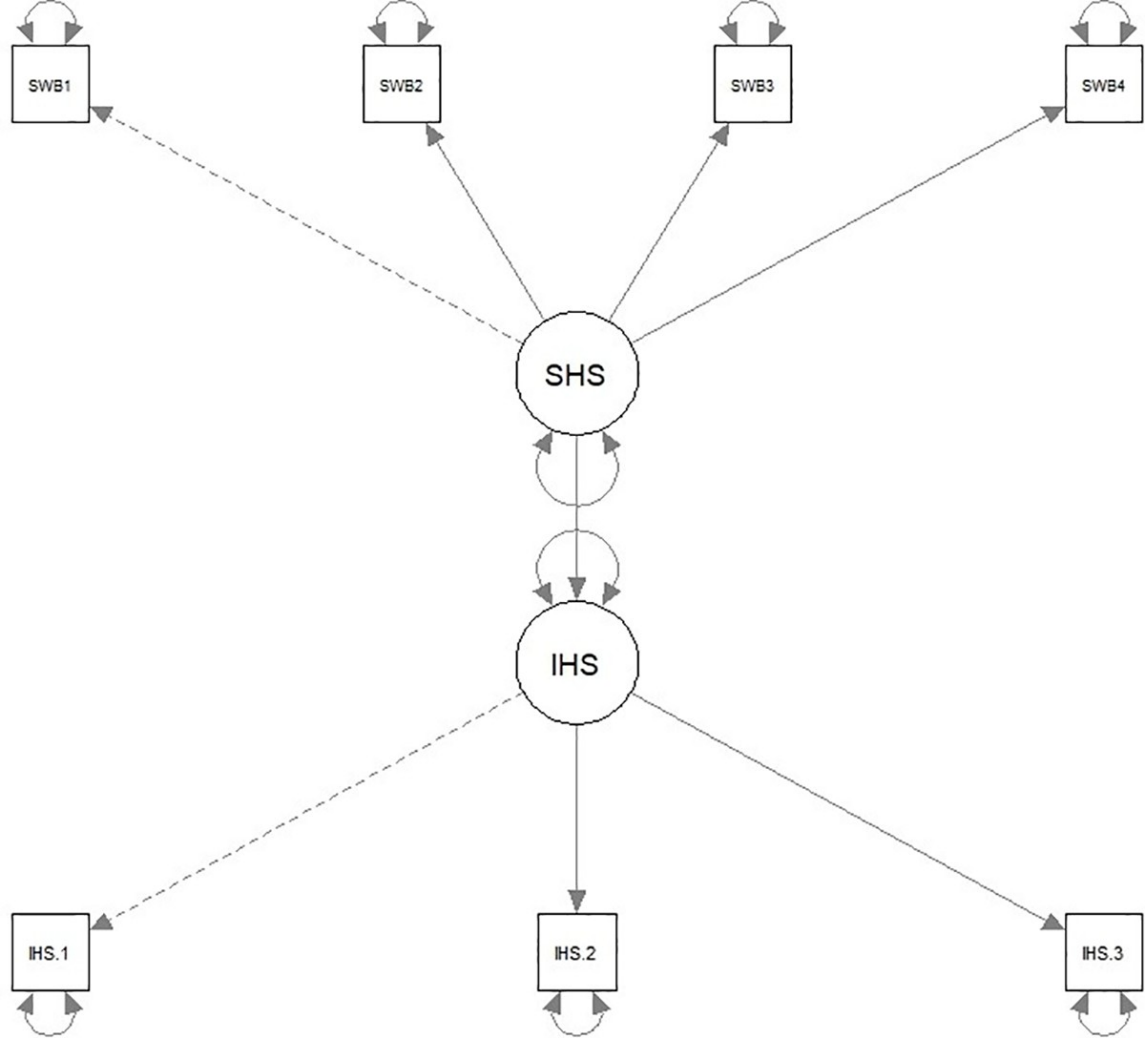
SEM model displaying the correlation between the happiness latent variables. *Note*. IHS = Interdependent Happiness Scale. SHS = Subjective Happiness Scale. Model fit statistics: RMSEA = .06, CFI = .98, *R*^*2*^ = .63. Estimate between SHS and IHS: *β* = .79, *b* = .31, *z* = 72.99, *p* < .001. IHS.1 was an average of the first 3 items on the IHS, IHS.2 was an average of the next 3 items on the IHS, and IHS.3 was an average of the last 3 items on the IHS.

A model with the two latent happiness variables was first fitted using all of the data (see Fig 1). The first factor loadings for each measure were fixed to 1 and the SHS was set as the predictor variable. Results indicated overall good fit for the model (RMSEA = .06, CFI = .98). Unsurprisingly, the SHS was significantly related to the IHS, *b* = .31, *β* = .79, *z* = 72.99, *p* < .001. Next, the same model was used to calculate the relationship between the SHS and the IHS within each country. Results are presented in Table 7. The countries with the strongest standardized relationship between the SHS and the IHS were Hungary (*β* = .97), New Zealand and Romania (*β* = .93). The countries with the weakest standardized relationship between the two happiness measures were Indonesia (*β* = .31) and Uganda (*β* = .36). Both Western and Eastern European countries had the highest average association between the happiness measures (*β*_*Mean*_ = .85) while the lowest associations were found in African countries (*β*_*Mean*_ = .66). Overall, while the relationship between the two happiness measures varied across countries, the were no countries in which the two measures were unrelated or negatively associated with each other.

**Table 7:** Results from structural equation model with IHS ~ SHS.

Country	*β*	*b*	*SE*	*R*^*2*^
Hungary	.97	.46	.04	.94
New Zealand	.93	.38	.04	.87
Romania	.93	.42	.04	.86
Belgium	.90	.40	.09	.81
Russia	.90	.36	.04	.81
Croatia	.90	.26	.03	.80
Peru	.89	.46	.06	.80
United Kingdom	.89	.37	.04	.79
Sweden	.89	.38	.05	.78
France	.88	.27	.03	.77
Netherlands	.87	.25	.02	.76
Czech Republic	.86	.27	.03	.74
Macedonia	.86	.22	.06	.74
Latvia	.85	.19	.03	.73
Turkey	.85	.27	.03	.73
Slovakia	.85	.40	.05	.72
Thailand	.85	.27	.03	.72
Jordan	.85	.42	.05	.72
Switzerland	.85	.29	.02	.72
Italy	.84	.34	.02	.71
Poland	.84	.26	.03	.71
Hong Kong	.84	.32	.04	.71
Brazil	.84	.35	.03	.71
Norway	.84	.30	.04	.71
Germany	.84	.29	.02	.70
Singapore	.84	.30	.03	.70
Spain	.83	.36	.03	.69
South Korea	.83	.34	.03	.68
Taiwan	.82	.30	.04	.68
South Africa	.82	.33	.03	.67
Bulgaria	.82	.31	.03	.67
Denmark	.82	.26	.03	.67
Georgia	.81	.28	.04	.66
Austria	.81	.32	.06	.66
Israel	.81	.45	.06	.66
Estonia	.81	.32	.03	.65
Vietnam	.81	.30	.04	.65
United States	.81	.32	.01	.65
Australia	.80	.27	.03	.64
Ukraine	.80	.38	.04	.63
Kenya	.79	.42	.06	.63
Serbia	.79	.31	.03	.62
Argentina	.78	.37	.06	.61
Canada	.78	.27	.03	.60
Slovenia	.77	.27	.04	.60
Palestine	.77	.28	.03	.59
Lithuania	.77	.30	.03	.59
India	.76	.39	.07	.58
Senegal	.76	.29	.03	.57
Chile	.75	.31	.03	.57
Portugal	.75	.19	.03	.56
China	.74	.28	.02	.54
Mexico	.73	.32	.04	.53
Japan	.72	.35	.04	.52
Philippines	.70	.29	.03	.48
Malaysia	.66	.33	.05	.44
Pakistan	.66	.20	.04	.43
Greece	.65	.25	.03	.43
Bolivia	.62	.32	.05	.38
Colombia	.56	.24	.04	.32
Nigeria	.56	.20	.04	.31
Uganda	.36	.11	.05	.13
Indonesia	.31	.06	.03	.09
**Average**	**.79**	**.31**	**.04**	**.64**

*Note*. Countries are listed from highest to lowest *β*.

### Country-level analyses

The second set of analyses were conducted on the country level, using the results presented previously as the input data (Tables 2, 3 & 5) as well as country-level data acquired from sources independent from this study (see S1 File for these country level scores). These country level analyses were conducted to help interpret the results previously discussed by attempting to find patterns in the results. This procedure is similar to Multilevel Modeling (MLM) that tests for group (Level 2) predictors of individual (Level 1) relationships. However, given that many of the relationships involve summaries of individuals within countries (e.g., reliability of a measure) rather than individual scores, we could not use the MLM framework for analyses. Fortunately, the large number of countries presented here allow for correlations to be conducted on the group level, with a total sample size ranging from 45 to 63 (countries).

#### Relationship between happiness measure reliabilities

The previous tests of reliability for the happiness measures resulted in multiple scores of internal consistency for each country and for each measure. We were interested to see if the same countries with good reliabilities for one happiness measure also produced good reliabilities for the other happiness measure. Correlations between the happiness measure reliabilities across countries were conducted for the general factor saturation, total common variance, smallest split-half reliability, and average communality scores for the items (see Table 8). There was a significant positive correlation between the two happiness measures for the total common variance *r*(61) = .34, *p* = .006, smallest split half reliability *r*(61) = .38, *p* = .002, and the average communality scores *r*(61) = .27, *p* = .03, but not for the general factor saturation *r*(61) = -.03, *p* = .82.

**Table 8:** Correlation between happiness measure reliabilities across countries.

		SHS			
		*ω*_*h*_	*ω*_*t*_	*β*	${\bar{h}}^{2}$
IHS	*ω*_*h*_	-.03			
	*ω*_*t*_		**.34**		
	*β*			**.38**	
	${\bar{h}}^{2}$				**.27**

*Note*. N = 63 countries. Correlations significant at the .05 level are **bolded**.

ω_t_ = total common variance, ω_h_ = general factor saturation, β = smallest split half reliability, ${\bar{h}}^{2}$ = average communality score.

#### Country-level predictors of happiness measure properties

**Fig 2:**
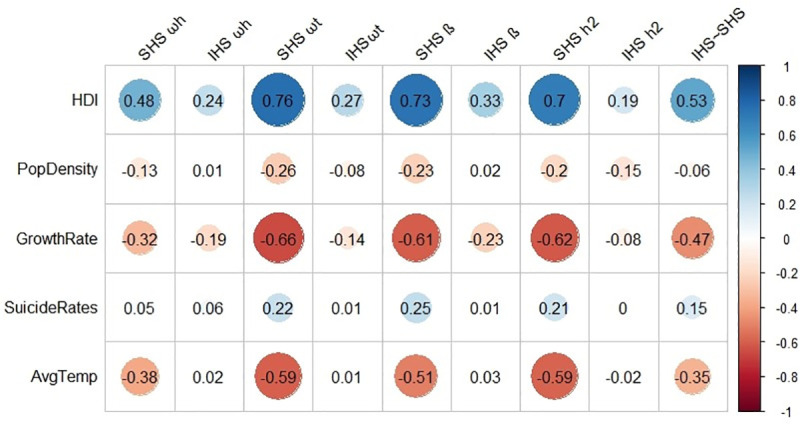
Country level correlations between objective country level variables and happiness variable reliabilities. *Note*. IHS = Interdependent Happiness Scale, SHS = Subjective Happiness Scale, ω*t* = total common variance, ω*h* = general factor saturation, β = smallest split half reliability, h2 = average communality score, HDI = Human Development Index, PopDensity = population density, GrowthRate = population growth rate, SuicideRate = suicide rate, AvgTemp = average daily temperature.

The last set of analyses attempted to find predictors of the happiness measure reliabilities and associations. If there are meaningful patterns in the data for the assessment of happiness across countries then these patterns can be predicted from other country-level variables. The first set of predictors were objective country level variables obtained from government sources and include the Human Development Index (HDI), population growth rate, population density, average suicide rate, and average temperature of a country. Full results are shown in Fig 2. Across these objective country level variables, the best predictor of happiness measure reliability was HDI, and these associations were higher for the SHS than the IHS. HDI was positively correlated with all four of the SHS reliabilities (general factor saturation *r*(60) = .48, *p* < .001, total common variance *r*(60) = .76, *p* < .001, lowest split half reliability *r*(60) = .73, *p* < .001, and average communality score *r*(60) = .70, *p* < .001). For the IHS, HDI was significantly correlated with two of the reliabilities (total common variance *r*(60) = .27, *p* = .03, lowest split half reliability *r*(60) = .33, *p* = .009). The population growth rate (*rω*_*h*_(60) = -.32, *rω*_*t*_(60) = -.66, *r*_*β*_ (60) = -.61, *r$\bar{h}$*^*2*^(60) = -.62) and average temperature (*rω*_*h*_(60) = -.38, *rω*_*t*_(60) = -.59, *r*_*β*_ (60) = -.51, *r$\bar{h}$*^*2*^(60) = -.59) of a country were negatively related to all of the SHS reliabilities but none of the IHS reliabilities. Suicide rates were unrelated to any of the happiness measure reliabilities. The strongest predictor of the correlation between the two happiness measures was a country’s HDI *r*(60) = .53, *p* < .001, population growth rate *r*(60) = -.47, *p* < .001, and average daily temperature *r*(60) = -.35, *p* = .005.

**Fig 3:**
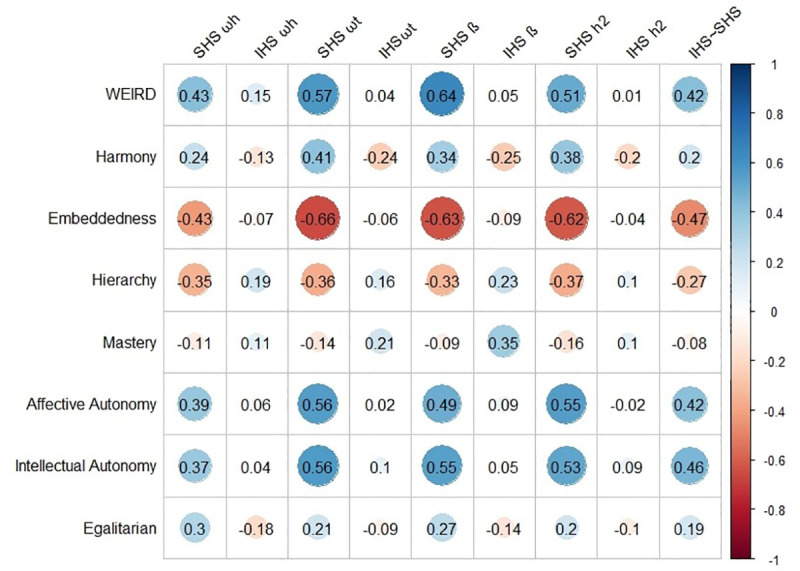
Country level correlations between subjective country level variables and happiness variable reliabilities. *Note*. IHS = Interdependent Happiness Scale, SHS = Subjective Happiness Scale, ω*t* = total common variance, ω*h* = general factor saturation, β = smallest split half reliability, h2 = average communality score. WEIRD scores originally from Muthukrishna et al. [37], values scores originally from Schwartz [38].

The correlations for subjective country level variables included a measure of WEIRDness and Schwartz’s values (see Fig 3). Consistent with the objective country level variables, there were many more correlates for the SHS reliabilities than the IHS reliabilities. For example, a country’s WEIRD score was positively correlated with the SHS general factor saturation *r*(43) = .43, *p* = .003, total common variance *r*(43) = .57, *p* < .001, lowest split half reliability *r*(43) = .64, *p* < .001, and average communality score *r*(43) = .51, *p* < .001 but unrelated to any of the IHS reliabilities. Additionally, countries with higher SHS reliabilities also scored higher on the values of Affective Autonomy (*rω*_*h*_(57) = .39, *rω*_*t*_(57) = .56, *r*_*β*_ (57) = .49, *r$\bar{h}$*^*2*^(57) = .55), and Intellectual Autonomy (*rω*_*h*_(57) = .37, *rω*_*t*_(57) = .56, *r*_*β*_ (57) = .55, *r$\bar{h}$*^*2*^(57) = .53) and lower on the value of Embeddedness (*rω*_*h*_(57) = -.43, *rω*_*t*_(57) = -.66, *r*_*β*_ (57) = -.63, *r$\bar{h}$*^*2*^(57) = -.58). Consistent with the objective country level correlates, there were substantially far fewer significant IHS reliability correlations. The only significant relationship was between the lowest split half IHS reliability and higher levels of valuing Mastery *r*(57) = .35, p = .007. This cultural value was unrelated to any of the SHS reliabilities. The strongest predictors of the correlation between the two happiness measures were a country’s WERID score *r*(43) = .42, p = .004, and the values of Intellectual *r*(43) = .46, *p* < .001 and Affective Autonomy *r*(43) = .42, *p* < .001 and less Embeddedness *r*(43) = -.47, *p* < .001.

## Discussion

### Reliability of the independent (SHS) and interdependent (IHS) measures of happiness

Multiple tests of measurement reliability revealed that, as might be expected, the reliability of each measure of happiness was stronger in regions more culturally similar to the country of the measure’s origin. Specifically, the interdependent measure of happiness had the highest overall reliabilities in East Asian countries, while the independent measure of happiness had the highest reliabilities in Western Europe. Interestingly, the reliabilities of the two measures of happiness were highly similar between the United States and Japan, the two countries in which the SHS and IHS measure were developed, respectively. As can be seen in Tables 2 and 3, the reliabilities of the measure of independent happiness were only slightly higher in the country of origin (the United States) than in Japan. The same held true for Japan, where the reliabilities of the measures of interdependent happiness were only slightly higher than the reliabilities in the United States. In both countries, the reliabilities of the measure of independent happiness were higher than the reliabilities of the measure of interdependent happiness, although this difference was less pronounced in Japan. While these subtle differences between the United States and Japan still align with theoretical predictions, differences in the reliabilities of the happiness measures become more notable when compared across the remaining 61 countries. For example, the interdependent measure of happiness performed much better than the independent measure of happiness in South Asian countries and the Middle Eastern countries. Additionally, the lowest reliabilities for both happiness measures were found in African countries, suggesting that neither conceptualization of happiness might be particularly well-suited for those cultures.

Additionally, the ranges of reliabilities across countries were different for the two happiness measures. The range of the reliabilities for the independent measure of happiness varied drastically while the range of reliabilities for the interdependent measure of happiness were much smaller. This discrepancy appeared despite the comparable overall averages in reliabilities across countries, with the SHS performing slightly better than the IHS overall. Thus, while the SHS has some of the highest reliabilities in certain countries (generally WEIRD ones), it also had some of the lowest reliabilities in other countries (generally non-WEIRD ones), while the reliabilities of the IHS varied less. Higher reliabilities of measures are generally considered better; however, for cross-cultural researchers interested in comparing measures across countries, the equivalence of a measure’s reliability maybe more important than its size, as variations in reliability can artificially inflate or deflate comparisons between countries [49]. Thus, despite the slightly lower overall reliability of the IHS than the SHS, we believe that in most cases the IHS would still be a better cross-cultural instrument.

The reliability of a measure is also a way to assess its coherence or “schema” in a particular culture. Higher reliabilities mean participants are responding to each item on the scale in a manner consistent with the putatively underlying latent trait, in this case happiness. A lower reliability suggests that the latent concept or structure of happiness is not as strong or coherent for that group, or that the items are assessing multiple aspects of happiness that do not map equally well onto the underlying construct. Treating reliabilities as an assessment of a construct’s coherence means that we can seek to predict the overall coherence of a measure across countries using other country-level data. It also suggests that it might be a mistake to “correct” the SHS for attenuation within countries where it has low reliability, since this may indicate that the SHS is a less appropriate measure in those countries and any corrections would only mask that fact.

The reliability of the SHS was related to many country-level variables, including economic development and a country’s “WEIRDness.” Specifically, coherence of the independent happiness measure was stronger in countries with higher development, less population growth, and in colder climates. Additionally, several cultural values were related to the reliabilities, or coherence, of the SHS. Greater coherence of the independent happiness measure was stronger in countries that value autonomy, both affective and intellectual. These countries value each individual’s uniqueness and, particularly for affective autonomy, “encourage individuals to pursue affectively positive experience for themselves” [53]. Additionally, the reliabilities for the SHS were lower in countries that value embeddedness with others, suggesting less interdependence in general as well as for defining one’s happiness. Thus, it appears that the concept of independent happiness is more coherent in the more developed, autonomous, WEIRD countries.

For interdependent happiness, there were far fewer country-level correlates with the reliabilities. However, given the more limited range of reliabilities compared to the SHS reliabilities as previously discussed, it is perhaps unsurprising that we were not able to find as many country level predictors. The IHS reliabilities were weakly related to a country’s economic development and higher in countries that value mastery over harmony. Interestingly, the reliability of the interdependent happiness measure was completely unrelated to a country’s “WEIRDness.” Thus, interdependent happiness may not be a WEIRD (or even non-WEIRD) construct; but rather be more uniformly meaningful across all countries. In that sense IHS may be a more “universal” measure of happiness than the SHS. This finding is consistent with previous work on cross-cultural differences in conceptual definitions of happiness. Delle Fave and colleagues [1] found that the most universal definition of happiness across 12 countries was harmony, a concept more commonly associated with the East Asian view on happiness, rather than the Western view. Thus, these universal lay definitions of happiness may explain why the IHS, developed in East Asia, performed more consistently across cultures than the SHS.

### Conceptual overlap between independent (SHS) and interdependent (IHS) happiness

Further analyses attempted to assess the degree of similarity between the two measures. Overall, the two measures of happiness were positively related to each other in every country assessed, however the strength of this relationship still varied cross-culturally. Individuals were more likely to associate these two measures of happiness in “WEIRDer” countries, i.e., those most similar to the United States. Additionally, the relationship between the two happiness measures was stronger in countries with more development, less population growth, and where people value more autonomy and less interdependence with others. These correlations are consistent with regional averages found in the data. Specifically, the strongest correlations between the two happiness measures were in European countries while somewhat lower in East Asian and Latin American countries. Interestingly, the lowest correlations between the happiness measures were found in Africa. Given that the African countries also had the lowest reliabilities for both happiness measures this suggests that the two measures may not only be more conceptually distinct in Africa but that neither measure may be fully appropriate for assessing happiness in that cultural context.

The two measures of happiness tested in this article originated from cultures with distinct historical roots and religious traditions [5]. The West has historically been influenced by a self-centered Protestant work ethic that defines happiness as a personal achievement and individuals as distinct, independent, and responsible for their own fate. In contrast, the Eastern ideologies of Buddhism, Taoism, and Confucianism emphasize the interconnectedness of everyone and everything, prioritizing harmony and balance over individual achievement [5]. Thus, it is perhaps unsurprising that both the American and Japanese measures of happiness performed worse in the regions lacking either Christian Protestant or Buddhist traditions (e.g., Africa and the Middle East) while generalizing better to Latin America, Europe, and the rest of Eastern Asia. The lower performance of both happiness measures in Africa and the Middle East further highlights the need for cross-cultural research to expand beyond the traditional East vs. West dichotomy (often limited even further to comparisons between Japan and the US). While it seems clear that the two measures of happiness presented here miss some aspect crucial to the cultures outside of the Eastern and Western contexts in which the measures were developed, it is less clear what these aspects are. To fill this gap in the literature remains an important next step for researchers interested in developing a universal measure of happiness.

### Limitations and future directions

The current study used country as a proxy for culture; however, country boundaries do not always correspond to cultural boundaries. Indeed, cultural boundaries are often extremely difficult to define, as numerous subcultures may exist within dominant cultures [50]. Thus, many researchers simplify or bypass the cultural definition problem by using country as the grouping variable. While using country as a proxy for culture far from a perfect solution, it does allow researchers to more easily compare results across studies. Additionally, it allows researchers to use country-level data, such as HDI, as predictors of individual level outcomes. This method is also of particular relevance to national governments interested in the well-being of their citizens.

Another potential limitation of the present study is the use of members of college communities as the primary source of participants. While data from non-college participants were also collected in a handful of countries, they were excluded from the present analyses to match the samples across countries and avoid confounding the results [51]. Because the vast majority of psychological studies use student participants [52], the results of this study are directly relevant to most research on happiness elsewhere in the literature. For example, the seven cultural dimensions used in the subjective country level correlations presented here were originally developed using college student and teacher samples [53], making the results directly comparable to those from the current study’s sample. Additionally, since the present analyses are not intended to address the mean level of well-being across nations, but rather how coherent the construct is in each culture, there is less reason to assume college students will differ drastically from the rest of the population [54]. If anything, college students should be “WEIRDer” than other people in their countries because they are more ‘E’ducated and often ‘R’icher. Thus, any differences that are found among countries are even more notable.

Lastly, the results from this study represent only a first step in the assessment of cross-cultural differences in happiness. While the evidence suggests that the interdependent measure of happiness is more consistently reliable across countries than the independent measure, the next step would be to establish how these differences in reliability translate into mean level differences and predictors of happiness across countries. However, we believe establishing the reliability of the measures across cultures represents an important first step for the broader goal of comparing happiness around the world.

## Conclusion

In many ways, the two happiness measures performed surprisingly similarly across countries, despite their conceptual and theoretical differences and different national origins. Around the world, individuals who were more likely to report being independently happy were also more likely to report being interdependently happy. However, methodological differences between the two measures still have important implications for the future study of happiness across cultures. Specifically, the reliability of the Interdependent Happiness Scale (IHS) performed more consistently across countries than the Subjective Happiness Scale (SHS). Additionally, the reliability of the IHS was less dependent upon country-level factors, such as the economic development of a country, in that sense making it a less “WEIRD” measure. Thus, cross-cultural researchers interested in incorporating a more universal measure of happiness should consider the Interdependent Happiness Scale as a useful tool for cross-cultural comparisons. Additionally, the weaker performances of both happiness measures in the Middle East and Africa point to the need for more research to expand beyond the traditional East vs. West dichotomy. Thus, while currently the IHS seems to be a better cross-cultural instrument than the SHS, future research should explore other emic measures of happiness developed in the Middle East and Africa that can provide a more universal and comprehensive definition of happiness.

What does it mean to be happy? The answer, the present study shows, indeed depends to an important degree on where you live.

## Supporting information

S1 File (DOCX)
