# Supplementary material for: Happiness around the world: A combined etic-emic approach across 63 countries
Source: PLoS One. 2020 Dec 9;15(12):e0242718. doi: 10.1371/journal.pone.0242718 (PMC7725360; doi:10.1371/journal.pone.0242718)
Supplement: S1 File — (DOCX) [file pone.0242718.s001.docx]

| Table A | | | | | |
| --- | --- | --- | --- | --- | --- |
| *Objective country level scores from external datasets* | | | | | |
| Country | HDI | Population Density | Growth Rate | Suicide Rate | Average Temperature |
| Argentina | .82 | 16 | .91 | 13.9 | 64 |
| Australia | .94 | 03 | 1.03 | 10.4 | 58 |
| Austria | .89 | 106 | .47 | 11.7 | 48 |
| Belgium | .89 | 374 | .70 | 16.1 | 51 |
| Bolivia | .67 | 10 | 1.51 | 20.5 | 45 |
| Brazil | .75 | 25 | .73 | 6.0 | 78 |
| Bulgaria | .80 | 66 | -.61 | 11.2 | 50 |
| Canada | .92 | 04 | .73 | 10.4 | 48 |
| Chile | .84 | 24 | .77 | 9.1 | 61 |
| China | .74 | 147 | .41 | 8.5 | 58 |
| Colombia | .73 | 44 | .99 | 6.0 | 56 |
| Croatia | .83 | 75 | -.50 | 12.1 | 51 |
| Czech Republic | .88 | 137 | .12 | 10.6 | 47 |
| Denmark | .92 | 136 | .22 | 9.1 | 47 |
| Estonia | .86 | 31 | -.57 | 14.9 | 41 |
| France | .90 | 122 | .39 | 12.3 | 55 |
| Georgia | .77 | 65 | -.02 | 5.3 | 55 |
| Germany | .93 | 237 | -.16 | 9.1 | 48 |
| Greece | .86 | 83 | -.06 | 3.2 | 66 |
| Hong Kong | .92 |  | .32 |  | 75 |
| Hungary | .84 | 108 | -.25 | 15.7 | 51 |
| India | .61 | 445 | 1.17 | 16.0 | 75 |
| Indonesia | .69 | 144 | .86 | 3.0 | 68 |
| Israel | .90 | 395 | 1.51 | 5.4 | 68 |
| Italy | .88 | 206 | .19 | 5.4 | 56 |
| Japan | .90 | 348 | -.21 | 15.4 | 59 |
| Jordan | .72 | 107 | 2.05 | 3.9 | 63 |
| Kenya | .55 | 85 | 1.69 | 10.5 | 67 |
| Latvia | .83 | 32 | -1.08 | 17.4 | 44 |
| Lithuania | .85 | 46 | -1.08 | 26.1 | 42 |
| Macedonia | .75 | 83 | .17 | 6.4 | 54 |
| Malaysia | .80 | 95 | 1.37 | 6.5 | 82 |
| Mexico | .76 | 66 | 1.12 | 5.0 | 65 |
| Netherlands | .92 | 505 | .39 | 9.4 | 49 |
| New Zealand | .91 | 18 | .79 | 12.3 | 55 |
| Nigeria | .53 | 204 | 2.43 | 15.1 | 78 |
| Norway | .95 | 14 | 1.01 | 9.3 | 44 |
| Pakistan | .53 | 251 | 1.43 | 2.5 | 76 |
| Palestine | .66 | 756 |  |  | 66 |
| Peru | .74 | 25 | .95 | 6.0 | 68 |
| Philippines | .68 | 347 | 1.57 | 3.8 | 82 |
| Poland | .85 | 124 | -.13 | 18.5 | 45 |
| Portugal | .84 | 113 | .04 | 8.5 | 63 |
| Romania | .80 | 86 | -.33 | 9.2 | 52 |
| Russia | .80 | 09 | -.08 | 17.9 | 39 |
| Senegal | .49 | 80 | 2.39 | 12.1 | 76 |
| Serbia | .78 | 81 | -.46 | 8.6 | 53 |
| Singapore | .92 |  | 1.82 | 9.9 | 82 |
| Slovakia | .84 | 113 | -.01 | 15.0 | 51 |
| Slovenia | .89 | 103 | -.31 | 24.1 | 48 |
| South Africa | .66 | 46 | .99 | 12.3 | 63 |
| South Korea | .90 | 526 | .48 | 11.8 | 55 |
| Spain | .88 | 78 | .78 | 6.0 | 55 |
| Sweden | .91 | 24 | .81 | 12.7 | 44 |
| Switzerland | .94 | 137 | .69 | 10.7 | 49 |
| Taiwan |  |  | .17 |  | 69 |
| Thailand | .74 | 135 | .30 | 12.7 | 84 |
| Turkey | .76 | 103 | .52 | 8.6 | 58 |
| Uganda | .49 | 207 | 3.20 | 12.6 | 68 |
| Ukraine | .74 | 78 | .52 | 16.6 | 45 |
| United Kingdom | .91 | 271 | -.41 | 7.4 | 51 |
| United States | .92 | 35 | .81 | 12.6 | 59 |
| Vietnam | .68 | 299 | .93 | 7.2 | 73 |
| *Note*. HDI = Human Development Index | | | | | |

| Table B | | | | | | | | |
| --- | --- | --- | --- | --- | --- | --- | --- | --- |
| *Subjective country level scores from external datasets* | | | | | | | | |
| Country | Embeddedness | Intellectual Autonomy | Affective Autonomy | Harmony | Egalitarianism | Hierarchy | Mastery | WEIRDness |
| Argentina | 3.52 | 4.34 | 3.73 | 3.98 | 4.96 | 2.10 | 3.92 | .93 |
| Australia | 3.59 | 4.35 | 3.86 | 3.99 | 4.79 | 2.29 | 3.97 | .97 |
| Austria | 3.11 | 4.90 | 4.29 | 4.31 | 4.89 | 1.75 | 3.92 |  |
| Belgium | 3.25 | 4.64 | 3.94 | 4.35 | 5.20 | 1.69 | 3.84 |  |
| Bolivia | 4.07 | 4.34 | 2.71 | 4.11 | 4.74 | 2.66 | 3.87 |  |
| Brazil | 3.62 | 4.27 | 3.52 | 4.03 | 4.89 | 2.37 | 3.93 | .93 |
| Bulgaria | 3.87 | 4.29 | 3.47 | 4.13 | 4.13 | 2.68 | 4.02 | .89 |
| Canada | 3.31 | 4.66 | 4.16 | 3.99 | 4.89 | 1.98 | 4.04 | .98 |
| Chile | 3.64 | 4.32 | 3.03 | 4.33 | 5.06 | 2.25 | 3.78 | .92 |
| China | 3.74 | 4.18 | 3.30 | 3.78 | 4.23 | 3.49 | 4.41 | .83 |
| Colombia | 3.86 | 4.30 | 3.61 | 3.66 | 4.69 | 2.90 | 4.03 | .89 |
| Croatia | 4.00 | 4.35 | 3.92 | 4.02 | 4.60 | 2.55 | 4.05 |  |
| Czech Republic | 3.59 | 4.62 | 3.49 | 4.27 | 4.45 | 2.22 | 3.75 |  |
| Denmark | 3.19 | 4.77 | 4.30 | 4.16 | 5.03 | 1.86 | 3.91 |  |
| Estonia | 3.81 | 4.23 | 3.36 | 4.31 | 4.58 | 2.04 | 3.79 | .88 |
| France | 3.20 | 5.13 | 4.39 | 4.21 | 5.05 | 2.21 | 3.72 | .92 |
| Georgia | 4.12 | 4.00 | 3.47 | 4.09 | 4.66 | 2.46 | 3.73 | .85 |
| Germany | 3.10 | 4.84 | 4.20 | 4.54 | 5.01 | 1.82 | 3.93 | .92 |
| Greece | 3.41 | 4.39 | 3.92 | 4.40 | 4.84 | 1.83 | 4.25 |  |
| Hong Kong | 3.76 | 4.28 | 3.20 | 3.50 | 4.50 | 2.91 | 4.08 | .90 |
| Hungary | 3.60 | 4.57 | 3.63 | 4.34 | 4.51 | 1.94 | 3.73 | .89 |
| India | 3.97 | 4.02 | 3.48 | 3.92 | 4.45 | 3.05 | 4.28 | .91 |
| Indonesia | 4.27 | 3.94 | 3.41 | 3.82 | 4.32 | 2.56 | 3.84 | .81 |
| Israel | 3.85 | 4.41 | 3.58 | 3.43 | 4.69 | 2.56 | 4.06 |  |
| Italy | 3.46 | 4.91 | 3.30 | 4.62 | 5.27 | 1.60 | 3.81 | .94 |
| Japan | 3.49 | 4.78 | 3.76 | 4.21 | 4.36 | 2.65 | 4.06 | .88 |
| Jordan | 4.20 | 4.05 | 3.36 | 3.67 | 4.40 | 2.50 | 4.20 | .80 |
| Kenya |  |  |  |  |  |  |  |  |
| Latvia | 3.83 | 4.22 | 3.48 | 4.46 | 4.32 | 1.80 | 3.75 |  |
| Lithuania |  |  |  |  |  |  |  |  |
| Macedonia | 3.91 | 4.24 | 3.01 | 4.03 | 4.40 | 2.72 | 4.00 |  |
| Malaysia | 4.35 | 4.15 | 2.98 | 3.65 | 4.41 | 2.25 | 3.91 | .87 |
| Mexico | 3.90 | 4.36 | 2.83 | 4.50 | 4.73 | 2.13 | 3.90 | .93 |
| Netherlands | 3.19 | 4.85 | 4.13 | 4.05 | 5.03 | 1.91 | 3.97 | .92 |
| New Zealand | 3.27 | 4.65 | 4.21 | 4.03 | 4.94 | 2.27 | 4.09 | .95 |
| Nigeria | 4.41 | 3.66 | 2.54 | 3.75 | 4.79 | 2.72 | 3.90 | .85 |
| Norway | 3.45 | 4.68 | 3.69 | 4.40 | 5.12 | 1.49 | 3.85 | .88 |
| Pakistan | 4.31 | 3.76 | 3.11 | 3.99 | 4.65 | 2.44 | 4.00 | .81 |
| Palestine |  |  |  |  |  |  |  | .85 |
| Peru | 3.92 | 4.30 | 2.98 | 3.71 | 4.84 | 2.76 | 4.08 | .90 |
| Philippines | 4.03 | 3.95 | 3.00 | 4.04 | 4.59 | 2.68 | 3.76 | .84 |
| Poland | 3.86 | 4.31 | 3.32 | 3.86 | 4.48 | 2.51 | 3.84 | .92 |
| Portugal | 3.43 | 4.53 | 3.62 | 4.27 | 5.21 | 1.89 | 4.11 |  |
| Romania | 3.78 | 4.61 | 3.45 | 4.11 | 4.48 | 2.00 | 4.06 | .89 |
| Russia | 3.81 | 4.30 | 3.51 | 3.90 | 4.38 | 2.72 | 3.96 | .91 |
| Senegal | 4.45 | 3.89 | 2.39 | 3.58 | 4.92 | 2.63 | 3.74 |  |
| Serbia | 3.57 | 4.72 | 3.70 | 3.96 | 4.44 | 1.61 | 4.03 | .93 |
| Singapore | 4.00 | 3.86 | 3.30 | 3.76 | 4.60 | 2.82 | 3.88 | .97 |
| Slovakia | 3.82 | 4.29 | 2.99 | 4.47 | 4.58 | 2.00 | 3.83 |  |
| Slovenia | 3.71 | 4.88 | 3.72 | 4.45 | 4.56 | 1.62 | 3.71 | .92 |
| South Africa | 4.03 | 3.85 | 3.48 | 3.86 | 4.52 | 2.59 | 3.89 | .92 |
| South Korea | 3.68 | 4.22 | 3.46 | 3.57 | 4.42 | 2.90 | 4.21 | .92 |
| Spain | 3.31 | 4.99 | 3.67 | 4.47 | 5.23 | 1.84 | 3.80 | .92 |
| Sweden | 3.12 | 5.09 | 4.24 | 4.46 | 4.90 | 1.83 | 3.81 | .89 |
| Switzerland | 3.19 | 4.99 | 4.29 | 4.17 | 4.99 | 2.24 | 3.86 | .94 |
| Taiwan | 3.82 | 4.36 | 3.27 | 4.12 | 4.31 | 2.69 | 4.00 | .90 |
| Thailand | 4.02 | 4.02 | 3.63 | 3.84 | 4.29 | 3.23 | 3.88 | .86 |
| Turkey | 3.77 | 4.45 | 3.37 | 4.23 | 4.77 | 2.97 | 3.98 | .87 |
| Uganda | 4.23 | 3.80 | 2.68 | 3.97 | 4.39 | 2.99 | 4.02 |  |
| Ukraine | 3.93 | 4.08 | 3.49 | 3.87 | 4.31 | 2.56 | 3.99 | .92 |
| United Kingdom | 3.34 | 4.62 | 4.26 | 3.91 | 4.92 | 2.33 | 4.01 |  |
| United States | 3.67 | 4.19 | 3.87 | 3.46 | 4.68 | 2.37 | 4.09 | 1.00 |
| Vietnam |  |  |  |  |  |  |  | .83 |
| *Note.* The first seven variables listed all come from Schwartz’s cultural values. WEIRDness indicates degree of psychological similarity to the United States (up to 1). | | | | | | | | |

| Table C | | | | | |
| --- | --- | --- | --- | --- | --- |
| *Country means for each happiness measure* | | | | | |
| Country | Total N | SHS | IHS | IHSpomp | SHSpomp |
| Argentina | 140 | 4.87 | 3.36 | 67.27 | 69.59 |
| Australia | 196 | 4.52 | 3.43 | 68.57 | 64.52 |
| Austria | 113 | 4.79 | 3.47 | 69.40 | 68.46 |
| Belgium | 50 | 4.58 | 3.57 | 71.47 | 65.43 |
| Bolivia | 135 | 4.58 | 3.24 | 64.77 | 65.42 |
| Brazil | 310 | 4.57 | 2.99 | 59.89 | 65.22 |
| Bulgaria | 152 | 4.92 | 3.50 | 70.09 | 70.28 |
| Canada | 304 | 4.76 | 3.54 | 70.82 | 68.00 |
| Chile | 386 | 4.94 | 3.49 | 69.70 | 70.60 |
| China | 432 | 4.89 | 3.73 | 74.57 | 69.87 |
| Colombia | 181 | 5.02 | 3.39 | 67.86 | 71.67 |
| Croatia | 218 | 4.68 | 3.59 | 71.74 | 66.91 |
| Czech Republic | 193 | 4.80 | 3.30 | 66.08 | 68.62 |
| Denmark | 246 | 5.03 | 3.67 | 73.32 | 71.86 |
| Estonia | 293 | 4.70 | 3.48 | 69.58 | 67.16 |
| France | 231 | 4.82 | 3.43 | 68.67 | 68.82 |
| Georgia | 140 | 4.69 | 3.32 | 66.37 | 67.02 |
| Germany | 458 | 4.68 | 3.34 | 66.87 | 66.87 |
| Greece | 225 | 4.81 | 3.34 | 66.80 | 68.73 |
| Hong Kong | 144 | 4.27 | 3.49 | 69.78 | 61.06 |
| Hungary | 178 | 4.88 | 3.39 | 67.72 | 69.74 |
| India | 221 | 5.18 | 3.68 | 73.63 | 73.97 |
| Indonesia | 131 | 4.78 | 3.64 | 72.74 | 68.29 |
| Israel | 173 | 5.22 | 3.54 | 70.87 | 74.61 |
| Italy | 717 | 4.51 | 3.21 | 64.21 | 64.40 |
| Japan | 243 | 4.66 | 3.26 | 65.27 | 66.50 |
| Jordan | 141 | 4.71 | 3.39 | 67.75 | 67.27 |
| Kenya | 139 | 5.35 | 3.52 | 70.33 | 76.39 |
| Latvia | 169 | 4.70 | 3.31 | 66.30 | 67.18 |
| Lithuania | 145 | 4.63 | 3.30 | 66.05 | 66.13 |
| Macedonia | 54 | 4.79 | 3.50 | 69.92 | 68.39 |
| Malaysia | 230 | 4.99 | 3.61 | 72.21 | 71.35 |
| Mexico | 247 | 5.35 | 3.71 | 74.27 | 76.49 |
| Netherlands | 301 | 4.72 | 3.57 | 71.45 | 67.38 |
| New Zealand | 129 | 4.69 | 3.52 | 70.30 | 66.97 |
| Nigeria | 135 | 5.37 | 3.67 | 73.43 | 76.77 |
| Norway | 159 | 4.72 | 3.58 | 71.67 | 67.41 |
| Pakistan | 114 | 4.83 | 3.46 | 69.12 | 68.98 |
| Palestine | 295 | 4.67 | 3.47 | 69.50 | 66.69 |
| Peru | 74 | 4.85 | 3.51 | 70.27 | 69.26 |
| Philippines | 337 | 4.66 | 3.30 | 66.02 | 66.54 |
| Poland | 234 | 4.51 | 3.38 | 67.50 | 64.45 |
| Portugal | 157 | 4.64 | 3.24 | 64.80 | 66.26 |
| Romania | 177 | 5.09 | 3.71 | 74.17 | 72.76 |
| Russia | 159 | 4.66 | 3.24 | 64.71 | 66.62 |
| Senegal | 635 | 4.89 | 3.58 | 71.64 | 69.92 |
| Serbia | 185 | 4.94 | 3.69 | 73.87 | 70.56 |
| Singapore | 136 | 4.37 | 3.46 | 69.17 | 62.45 |
| Slovakia | 148 | 4.66 | 3.45 | 68.92 | 66.63 |
| Slovenia | 123 | 4.70 | 3.61 | 72.12 | 67.13 |
| South Africa | 256 | 4.73 | 3.30 | 65.95 | 67.59 |
| South Korea | 281 | 4.56 | 3.54 | 70.84 | 65.20 |
| Spain | 419 | 4.72 | 3.49 | 69.75 | 67.43 |
| Sweden | 130 | 4.28 | 3.24 | 64.72 | 61.18 |
| Switzerland | 755 | 4.82 | 3.52 | 70.37 | 68.90 |
| Taiwan | 162 | 4.42 | 3.57 | 71.30 | 63.14 |
| Thailand | 196 | 4.86 | 3.55 | 71.00 | 69.48 |
| Turkey | 329 | 4.47 | 3.56 | 71.27 | 63.93 |
| Uganda | 93 | 5.12 | 3.26 | 65.23 | 73.08 |
| Ukraine | 244 | 5.01 | 3.35 | 67.01 | 71.50 |
| United Kingdom | 136 | 4.67 | 3.44 | 68.81 | 66.73 |
| United States | 1366 | 4.84 | 3.49 | 69.90 | 69.20 |
| Vietnam | 168 | 4.92 | 3.44 | 68.88 | 70.34 |
| **Average** | | **4.78** | **3.46** | **69.18** | **68.27** |
| *Note.* SHS = Subjective Happiness Scale, IHS = Interdependent Happiness Scale. Because the SHS is on a 7 point scale and the IHS is on a 5 point scale, POMP (percent of maximum possible) scores for both measures are included for comparability. | | | | | |
